# Appropriate use of inhaled nitric oxide in line with sustainable development goals

**DOI:** 10.1186/s40981-024-00752-x

**Published:** 2024-10-28

**Authors:** Keisuke Yoshida, Fumika Kawamata, Takayuki Hasegawa, Taichi Shiraishi, Satoki Inoue

**Affiliations:** 1 Department of Anesthesiology, Fukushima Medical University School of Medicine, 1 Hikarigaoka, Fukushima City, Fukushima, 960-1295 Japan; 2 Department of Anesthesiology, Southern Tohoku General Hospital, Koriyama, Fukushima Japan

To the Editor,

Inhaled nitric oxide (iNO) is a selective pulmonary vasodilator with the advantage of reducing pulmonary vascular resistance and improving ventilation/perfusion (V/Q) mismatch [1]. Because iNO does not cause hypotensive side effects in the systemic circulation, it is widely used for both approved and off-label indications in acute medical care, including managing perioperative pulmonary hypertension in pediatric/adult cardiac surgery, acute right heart failure during mechanical circulatory support [2], and hypoxemia associated with one-lung ventilation or respiratory disease. Some institutions use iNO routinely during/after cardiac surgeries.

On the other hand, there are several issues regarding iNO therapy. First, the evidence supporting its active clinical use remains limited. A recent meta-analysis showed that the effect of iNO on mortality was not statistically significant compared to standard care or placebo in cardiac surgery (relative risk 1.01, 95% credibility intervals 0.03–41) [3]. In addition, recent studies have reported that iNO reduced the duration of mechanical ventilation in cardiac surgery and improved oxygenation in hypoxemia due to COVID-19, but neither had any effect on mortality [4, 5]. Overall, there is limited evidence that iNO has a significant impact on clinical outcomes, and no evidence of benefit in terms of mortality [6]. Second, iNO can cause methemoglobinemia, which is a less common side effect but may be overlooked with normal oxygen saturation measurements [7]. Third, iNO therapy poses environmental pollution concerns. NO and NO_2_ produced by iNO therapy are nitrogen oxides (NOx), which are part of the causes of photochemical air pollution. Fourth, the cost of iNO therapy is estimated to be $220 per hour [3], and the issue of cost-effectiveness cannot be ignored. It is also important to note that the onset of action of iNO is extremely rapid (5–10 s) [3].

Taking into aforementioned drawbacks, routine use of iNO should be strictly avoided, and its indications should be evaluated on a case-by-case basis. Specifically, because the effects of iNO appear very rapidly, it is reasonable to use iNO for therapeutic purposes after signs of right heart failure are confirmed/evaluated via pulmonary artery catheter and/or echocardiography, or when severe hypoxemia possibly associated with V/Q mismatch is observed, rather than routine prophylactic administration in advance. Furthermore, the following points should always be considered during iNO therapy: (1) titrate iNO to the minimum effective dose; (2) measure NOx taking into account environmental pollution; (3) check methemoglobin regularly by using a CO-oximeter rather than a traditional pulse oximeter [7]; and (4) minimize fresh gas flow and leakage when using iNO in patients without tracheal intubation (i.e., via high-flow nasal cannula or non-invasive positive pressure ventilation). Daily evaluation of the feasibility of weaning from mechanical ventilation (spontaneous breathing trial) is known to contribute to the shortening of mechanical ventilation duration. Similarly, once iNO has been initiated, periodical evaluation of the feasibility of weaning from iNO should be done, although further research is needed to determine which iNO weaning protocols are optimal in the perioperative period of cardiac surgery.

## Data Availability

Not applicable.
